# Investigation of CD47 Expression in Renal Cell Tumors and Evaluation of Its Relationship with Prognostic Parameters

**DOI:** 10.3390/diagnostics15010053

**Published:** 2024-12-28

**Authors:** Ömer Faruk Dizibüyük, Zehra Bozdağ, Metin Karakök

**Affiliations:** 1 Department of Pathology, Cengiz Gokcek Maternity and Child Diseases Hospital, 27010 Gaziantep, Turkey; 2 Department of Pathology, Inonu University, Turgut Ozal Medical Center, 44280 Malatya, Turkey; zbozdagmd@gmail.com; 3 Department of Pathology, Faculty of Medicine, Gaziantep University, 27410 Gaziatep, Turkey; karakokmetin@yahoo.com.tr

**Keywords:** benign and malignant renal neoplasm, CD47 expression, prognosis, IHC

## Abstract

**Background/Objectives:** Renal cell carcinoma is an aggressive form of kidney cancer, contributing to an estimated 138,000 deaths globally in 2017. Traditional treatments like chemotherapy and radiation are generally considered ineffective. Additionally, CD47 has been identified as a crucial tumor antigen involved in the development and progression of various cancers, including renal cell carcinoma. The interaction of CD47 with SIRPα triggers a “don’t eat me” signal to the macrophages, inhibiting phagocytosis. Much progress has been made in targeting CD47 for cancer immunotherapy in solid tumors (STs) and hematological malignancies. This study aimed to evaluate CD47 expression in malignant and benign renal cell tumors and compare it with prognostic histopathological parameters. **Methods:** We included 160 malignant and 26 benign tumors. The malignant tumors consisted of renal cell carcinoma (RCC) subtypes including 37 clear cell, 30 chromophobe, 30 papillary type 1, 29 papillary type 2, and 34 unclassified RCC cases. As for the benign tumors, we included 26 oncocytoma cases. All samples were stained with anti-CD47 antibodies by immunohistochemistry methods. **Results:** The statistical analysis yielded a significant correlation between CD47 expression and survival, metastasis, and capsule invasion for the unclassified RCC cases. We did not find any further significant correlation between CD47 expression and the studied parameters. **Conclusions:** To the best of our knowledge, our study is the first to research CD47 expression in benign and malignant renal carcinoma subtypes. Further large-scale studies are needed to determine the expression profile of CD47 in renal cell tumors.

## 1. Introduction

The term renal cell carcinoma (RCC) describes a group of tumors originating from the renal tubules, with distinct morphological and genetic characteristics. Kidney cancer accounts for approximately 2% of all cancer diagnoses and cancer deaths worldwide, with incidence rates generally higher in developed countries. It is twice as common in men as in women. Globally, RCC is the 16th leading cause of cancer-related deaths, with mortality rates increasing by approximately 1.5–5.9% annually. Although it is most frequently observed in patients in their 6th to 7th decade of life, it is not uncommon in younger patients, with a 10% incidence in those under the age of 45 [1,2,3].

In approximately 30% of the cases, the carcinogenic effects of smoking have been held responsible. Obesity and type 2 diabetes mellitus are risk factors, particularly in women. Besides these instances, although several risk factors such as cadmium and petroleum products have been identified, the etiology remains unclear in most cases [4,5,6].

Approximately 25–30% of the patients with renal cell carcinoma present with metastatic tumors, and 40% die from the disease [7]. Approximately 30–50% of the localized cases exhibit recurrence or metastasis [8,9].

The most important parameter determining the prognosis is the histopathological subtype of the tumor, while tumor size, histological grade, pathological stage, capsular invasion, lymphovascular invasion, and the presence of metastasis are other prognostic factors [10].

There are numerous studies on the prognosis of RCC. Some of these focus on the biological and histological characteristics of the tumor, while others examine its molecular features. Although certain aspects of prognosis have become clearer because of these studies, there are still unresolved points regarding the pathogenesis of RCC.

CD47, also known as integrin-associated protein (IAP), is a cell surface protein and a member of the immunoglobulin family [11,12]. CD47 functions as a receptor for integrins and thrombospondin-1 (TSP-1) and plays a significant role in various biological processes such as immune cell activation, cell migration, and neural development [13,14,15,16,17]. This protein, normally found on the plasma membrane of all hematopoietic cells and many other cell types, plays a crucial role in preventing the phagocytosis of normal cells by macrophages [18,19,20,21]. The CD47 protein is highly expressed in various types of cancers, including acute and chronic myeloid leukemia, non-Hodgkin lymphomas, multiple myeloma, leiomyosarcoma, glioblastoma, and carcinomas of the bladder, ovary, prostate, breast, and colon. Studies have found that its increased expression is associated with enhanced anti-phagocytic potential of the tumor cells and poor prognosis [22,23]. In this study, we aimed to evaluate the correlation of CD47 expression with prognostic parameters (tumor type, tumor size, histological grade, pathological stage, capsular invasion, lymphovascular invasion, and the presence of metastasis) in cases of benign and malignant renal cell tumors.

## 2. Materials and Methods

### 2.1. Patients

By reviewing archival records, a total of 160 patients who underwent surgery for renal cell carcinoma and 26 patients diagnosed with oncocytoma at Gaziantep University Medical Faculty Hospital, Türkiye, between 2003 and 2016 were included in the study. The surgical procedures consisted in radical nephrectomy in 164 patients and simple nephrectomy in 22 patients. Among the renal cell carcinoma cases, 37 were clear cell RCC, 30 were chromophobe RCC, 30 were type 1 papillary RCC, 29 were type 2 papillary RCC, and 34 were unclassified RCC cases. All H&E-stained preparations from these cases were reevaluated based on the 2016 WHO classification.

The cases were retrospectively analyzed in relation to age, sex, type of surgery, histological subtype and grade, pathological tumor stage, and the presence of capsule, Gerota’s fascia, perirenal adipose tissue, renal vein invasion, lymphovascular invasion, lymph node metastasis, and distant organ metastasis with respect to the 5-year survival. Suitable paraffin blocks containing adequate tumor tissue and free from tissue-tracking artifacts were selected for immunohistochemical studies.

### 2.2. Immunohistochemical Staining

Sections of 4 microns in thickness obtained from the selected paraffin blocks for immunohistochemical studies were placed on poly-L-lysine-coated slides. The slides were first incubated at 37 °C for 15 min. Subsequently, immunohistochemical staining was performed using an anti-CD47 monoclonal antibody (Santa Cruz, Biotechnology, Dallas, TX, USA—anti-CD47 antibody, sc-12730 clone, 1:50 dilution) in an automatic staining device (Ventana Bench Mark Ultra, Roche Diagnostics, Rotkreuz, Switzerland, SN:316054). All stained sections were evaluated for staining prevalence and intensity by two different pathologists under an Olympus BX46 light microscope.

In the evaluation of CD47 expression by immunohistochemistry, the preparations were assessed for expression prevalence and intensity in tumor cells. Cells with membranous and cytoplasmic expression were considered positive. The endothelial cells served as internal controls, while cases of acute lymphoblastic/myeloid leukemia and urothelial carcinoma were used as external controls. The degree and intensity of expression were evaluated based on studies of CD47 in the literature. The absence of expression was scored as negative (0 = 0); the presence of expression in less than 10% of the cells was scored as 1+ (1+ ≤ 10%); the presence of expression in 10–25% of the cells was scored as 2+ (2+ = 10–25%); the presence of expression in 26–50% of the cells was scored as 3+ (3+ = 26–50%); and the presence of expression in more than 50% of the cells was scored as 4+ (4+ ≥ 50%). The intensity of expression in tumor tissue was scored on a scale of 0 to 3. Accordingly, the absence of expression was scored as 0, weak intensity as 1+, moderate intensity as 2+, and strong expression as 3+. The H score for each case was obtained by multiplying these two parameters (expression prevalence and intensity). An H score of <6 was considered low expression, while an H score of ≥6 was considered high expression.

### 2.3. Data Analysis

Descriptive statistics are presented as counts and percentages. The Chi-square test was used to compare categorical data. The analyses were conducted using the SPSS Windows 22.0 software package. A *p*-value of <0.05 was considered statistically significant.

## 3. Results

The patients’ characteristics are summarized in Table 1. Out of a total of 186 cases, weak CD47 expression was observed in 54 cases (29%), strong expression was found in 3 cases (1.6%), while no staining was noted in 129 cases (69.4%). Among the cases with strong staining, one belonged to the type 2 papillary RCC group, one to the chromophobe RCC group, and one was an unclassified RCC.

The clear cell RCC group showed weak expression in 3 of the 37 examined cases and no expression in 34 cases. The chromophobe RCC group showed strong expression in 1 of the 30 cases, weak expression in 23 cases, and no expression in 6 cases. None of the 30 papillary type 1 RCC cases showed CD47 expression. The papillary type 2 RCC group showed strong expression in 1 of the examined 30 cases, weak expression in 6 cases, and no expression in 21 cases. The oncocytoma group showed weak expression in 5 of the 28 cases and no expression 21 cases. The unclassified RCC group showed strong expression in 1 of the 34 cases, weak expression in 17 cases, and no expression in 16 cases.

While strong CD47 expression was not detected in any of the clear cell RCC cases, weak expression was observed in three of them. In 34 cases, no expression was observed. None of the patients in the oncocytoma group showed strong expression, while five patients showed weak expression. In 21 of the patients, no expression was detected. In 1 case of chromophobe RCC, strong expression was observed, while weak expression was noted in 23 cases. Expression was not detected in six cases. No staining was observed in any of the papillary type 1 clear cell RCC cases, while strong expression was found in one case of papillary type 2 clear cell RCC, and weak expression in six cases. Staining was not noted in 22 cases of papillary type 2 RCC. In 1 case of unclassified RCC, strong expression was observed, while weak expression was noted in 17 cases. Staining was not observed in 16 cases.

The immunohistochemical staining results are summarized in Table 2 and Table 3.

When comparing CD47 expression in relation to the pathological tumor stage, in the oncocytoma group, 24 cases were in the T1 stage, and 2 cases were in the T2 stage. There were no patients in the T3 and T4 stages in this group. In the papillary type 1 RCC group, no staining with the anti-CD47 antibody was observed in any of the patients. Therefore, a comparison of CD47 expression in relation to the pathological tumor stage could not be made between these two groups. However, among the other RCC subgroups, no statistically significant relationship was found between CD47 staining and pathological tumor stage. A significant relationship was found between the groups regarding CD47 expression overall (*p* = 0.001).

In chromophobe RCC and unclassified RCC cases, CD47 expression was significantly higher compared to that in the other groups. A significant relationship was found between chromophobe RCC and unclassified RCC cases regarding CD47 positivity (*p* = 0.024).

Among the 24 chromophobe RCC cases where CD47 expression was detected, lymphovascular invasion was identified in 3 cases, while no staining was observed in any of the clear cell RCC and papillary type 2 RCC cases. No statistically significant relationship was found between lymphovascular invasion and CD47 expression (*p* = 0.245).

In our study, it was determined that 6 of the 24 chromophobe RCC patients with CD47 staining, 5 of the 7 papillary type 2 RCC carcinoma patients, all 3 clear cell RCC patients, and 14 of the 18 unclassified RCC patients died within a follow-up period of less than 5 years. A statistically significant relationship was found between the expression rate of CD47 and survival time in the unclassified RCC cases (*p* = 0.001).

When evaluating the relationship between CD47 expression and capsule invasion, a significant relationship was found between CD47 expression and capsule invasion in the unclassified RCC cases, while no statistically significant difference was observed in the other groups.

All of the 30 patients with papillary type 1 RCC had localized tumors. In addition, 26 of the 29 patients with papillary type 2 RCC had localized tumors, and 3 had metastatic tumors. We also found that 24 of the 30 patients with chromophobe RCC had localized tumors, and 6 had metastatic tumors, while 18 of the 34 patients with unclassified RCC had localized tumors, and 6 had metastatic tumors.

**Figure 1:**
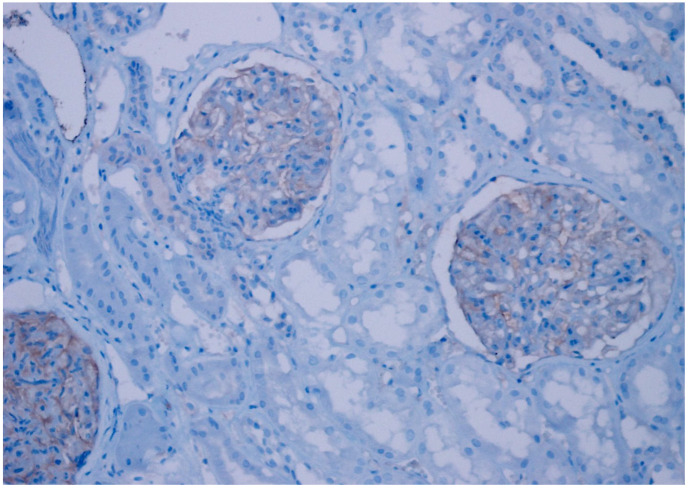
Positive staining of glomerular endothelium with anti-CD47 antibody (×100).

**Figure 2:**
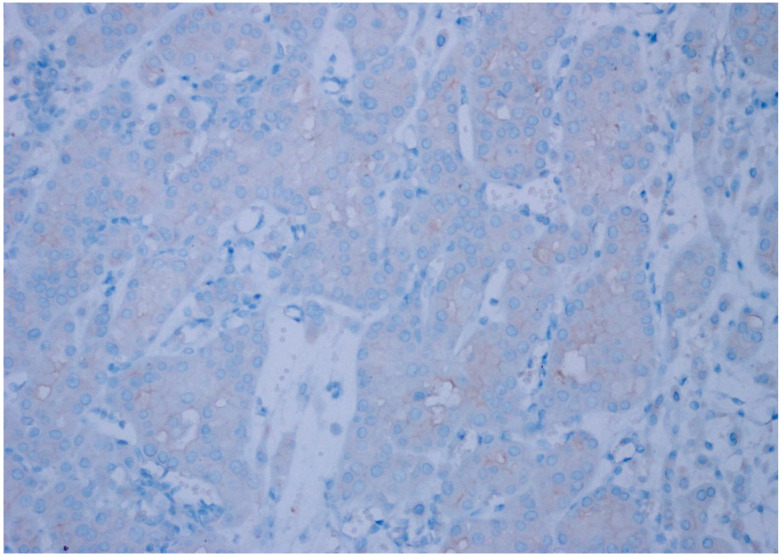
Weak intensity, 2+ expression (%10–25 prevalence) in oncocytoma, (CD47, ×200).

**Figure 3:**
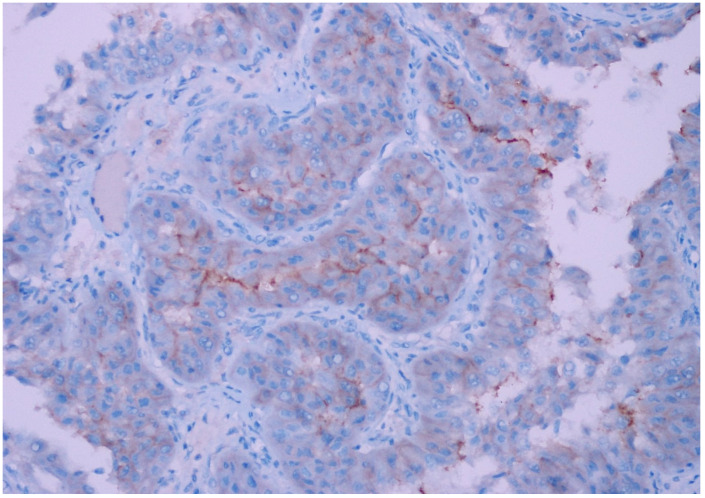
Moderate intensity, 2+ expression (10–25% prevalence) in papillary type 2 RCC, (CD47, ×200).

**Figure 4:**
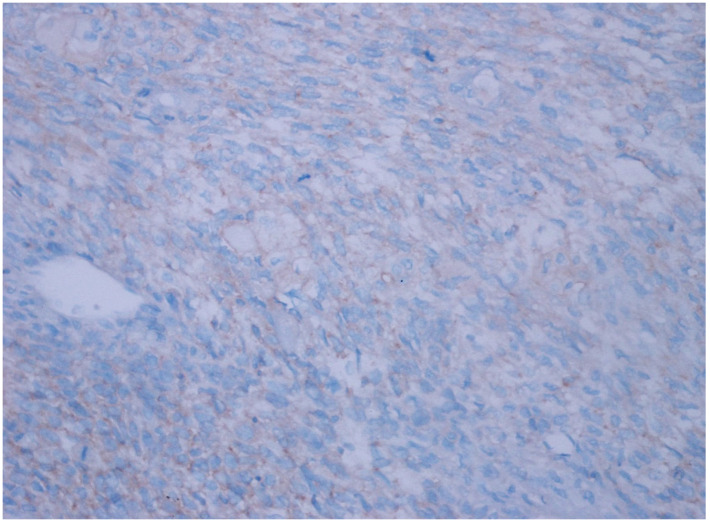
Weak intensity, 3+ expression (26–50% prevalence) in unclassified RCC, (CD47 ×200).

**Figure 5:**
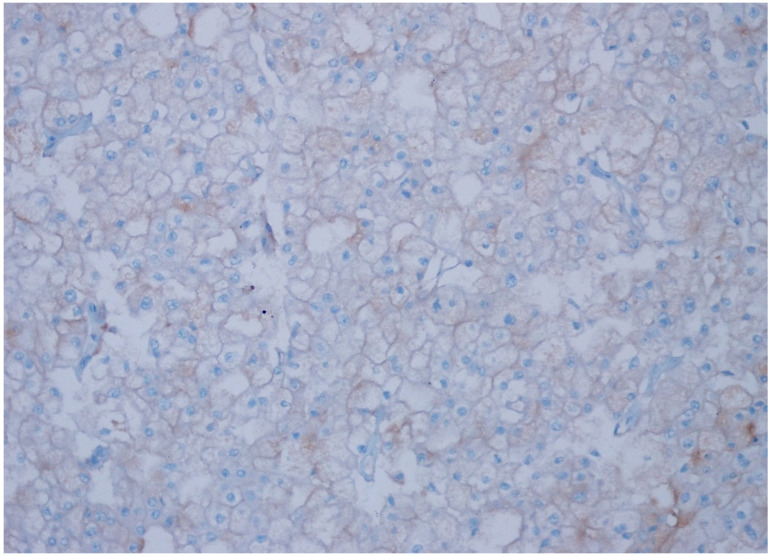
Weak intensity, 4+ expression (>50% prevalence) in chromophobe RCC, (CD47 ×200).

**Figure 6:**
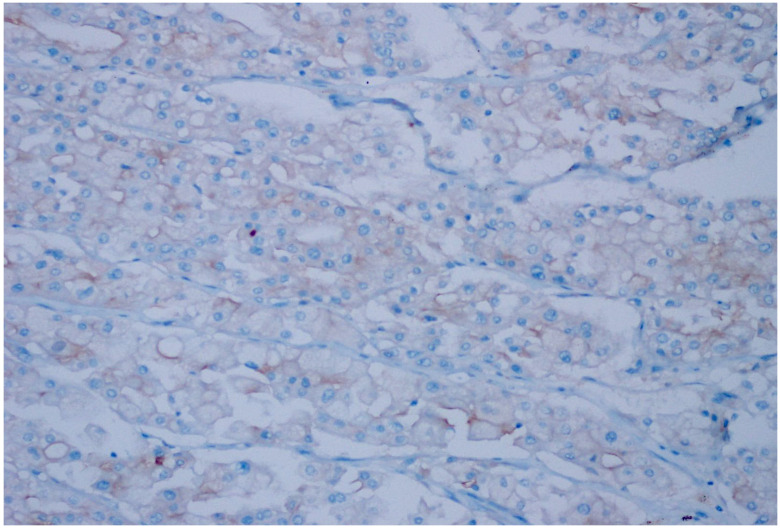
Weak intensity, 1+ expression (<10% prevalence) in clear cell RCC, (CD47 ×200).

In our study, metastasis was detected in 6 out of 24 chromophobe RCC patients with CD47 staining, in 1 out of 7 patients with papillary type 2 RCC, in 2 out of 3 patients with clear cell RCC, and in 16 out of 18 unclassified RCC cases. A significant difference was found between CD47 expression and metastasis in the unclassified RCC cases (*p* = 0.001). No statistically significant difference was observed in the other groups (Figure 1, Figure 2, Figure 3, Figure 4, Figure 5 and Figure 6).

## 4. Discussion

The binding of the CD47-SIRPa complex to macrophages creates a signal that negatively regulates macrophage activation and phagocytosis, inhibiting these functions in macrophages. Additionally, CD47 initiates heterotrimeric G-protein signaling along with signaling by members of the β1, β2, and β3 integrin family. Thus, CD47 modulates cell movement, leukocyte adhesion, cell migration, and phagocytosis [24].

The CD47 protein is highly expressed in many types of cancer, including acute and chronic myeloid leukemia, non-Hodgkin lymphomas, multiple myeloma, leiomyosarcoma, glioblastoma, and bladder, ovarian, prostate, breast, and colon carcinomas. Studies have found that increased CD47 expression is associated with enhanced anti-phagocytic potential of the tumor cells and poor prognosis [22,23,25,26,27,28].

There are studies in the literature that evaluate CD47 expression not only in solid tumors but also in precancerous lesions and invasive carcinomas. In their study, Xiaoying et al. [29] evaluated CD47 expression in cases of oral squamous cell carcinoma, normal oral mucosa, and low-risk oral leukoplakia and found significantly increased CD47 expression in the carcinoma cases.

In their study researching CD47 expression in primary cutaneous squamous cell carcinoma (SCC) and precursor lesions, Akel et al. [30] found significantly increased CD47 expression in SCCs compared to actinic keratosis (AK), keratoacanthoma, and in situ SCC (ISCC) lesions. The progressively increasing CD47 expression across the AK-ISCC-SCC spectrum suggests that CD47 expression may play a role in the progression from in situ malignancy to overt invasive carcinoma.

Edris et al. demonstrated in their study that CD47 is expressed at higher levels in leiomyosarcoma tumor cells compared to leiomyoma cells and that in vitro anti-CD47 monoclonal antibodies enhance macrophage-mediated phagocytosis in leiomyosarcoma cells [22].

In our series consisting of RCC cases exhibiting significant malignant behavior, excluding oncocytomas, CD47 expression was detected in 57 out of 186 cases (30.6%), while expression was not observed in 129 cases (69.4%). Among the cases showing positive expression, 24 (80%) were chromophobe RCC, 18 (53%) were unclassified RCC, 7 (24%) were papillary type 2 RCC, 5 (19%) were oncocytoma, and 3 (8%) were clear cell RCC cases. No expression was detected in the papillary type 1 RCC cases.

Park et al. [31] studied CD47 expression in 235 patients with clear cell RCC and found positive CD47 expression in 11.9% of the patients. This CD47 expression was associated with an aggressive phenotype and poor prognosis. In our study, we detected CD47 expression in 8% of 37 patients with clear cell RCC. Our study did not find a significant relationship between CD47 expression and poor survival in the clear cell RCC cases.

In our study, CD47 expression in the chromophobe RCC and unclassified RCC cases was significantly higher than in the other groups (*p* = 0.001). A significant relationship was found regarding CD47 positivity between chromophobe RCC and unclassified RCC cases (*p* = 0.024).

Renal oncocytomas account for approximately 5–9% of all kidney tumors. In oncocytomas, lesion extension beyond the kidney and into the surrounding adipose tissue can be observed, and in rare cases, invasion of the renal vein may also occur [32]. In our study, CD47 expression was detected in the oncocytoma cases, although not at a very high rate. This finding indicates that CD47 expression may occur in different histological subtypes of kidney tumors.

Papillary RCC is the second most common type among the RCC variants. Papillary RCC has a better prognosis compared to other RCC types. Papillary RCC is further divided into two subgroups: type 1 and type 2. The prognosis of type 2 is worse than that of type [33]. In our study, no staining was observed in papillary type 1 RCC cases, while an increased CD47 expression was detected in type 2 cases. When comparing the two types, the increased expression of CD47 in papillary type 2 RCC supports the idea that CD47 expression is a prognostic parameter.

Chromophobe RCCs constitute 5% of all RCCs. Compared to clear cell RCC, chromophobe RCC has a better prognosis [34]. In our study, the highest level of CD47 expression was detected in chromophobe RCC. This finding does not support the relationship between CD47 expression and prognosis.

Unclassified RCCs encompass cases that are included in the 2016 WHO classification but do not fit any of the RCC subtypes. They account for less than 5% of all kidney tumors. The mortality rate is 1.7 times higher than that of clear cell RCC [35]. In our study, CD47 expression was detected in more than half of the unclassified RCC cases. This result supports the relationship between CD47 expression and prognosis when compared to clear cell RCC cases, which showed lower CD47 expression. However, the presence of a statistically significant difference in CD47 expression between chromophobe RCCs and unclassified RCC cases does not support the relationship between CD47 expression and prognosis.

In their study on CD47 expression in patients with malignant melanoma, Fu et al. [36] demonstrated that tumors in advanced TNM stages (III–IV) had a higher level of CD47 expression compared to tumors in lower TNM stages (I–II). Zhao et al. [37] found in their study of tumor tissues from cases of non-small cell lung carcinoma that increased CD47 expression was significantly associated with T classification, clinical stage, lymph node metastasis, and distant metastasis.

In our study, when comparing the relationship between CD47 expression and pathological tumor stage, 24 cases in the oncocytoma group were classified as T1, and 2 cases as T2. There were no patients with T3 or T4 stage tumors in this group. In the papillary type 1 RCC group, no CD47 staining was detected in any of the patients. Therefore, no comparison could be made between these two groups regarding CD47 staining and pathological tumor stage. However, among the other RCC subgroups, no statistically significant relationship was found between CD47 staining and pathological tumor stage.

In our study, lymphovascular invasion was detected in 3 out of 24 chromophobe RCC cases with CD47 expression, while no CD47 expression was observed in clear cell RCC and papillary type 2 RCC cases with lymphovascular invasion. No statistically significant relationship was found between lymphovascular invasion and CD47 expression (*p* = 0.245). When evaluating the relationship between CD47 expression and capsule invasion, a significant relationship was found between CD47 expression and capsule invasion in the unclassified RCC cases, while no statistically significant difference was observed in the other groups.

In their study, Sudo et al. [38] observed a statistically significant relationship between CD47 expression in the primary tumor and clinicopathological factors in patients with gastric carcinoma. Yoshida et al. [39] reported that the survival rates of gastric cancer patients expressing CD47 immunohistochemically were significantly worse than those of CD47-negative gastric cancer patients. In another study, it was found that the prognosis of breast cancer patients with high levels of CD47 expression was significantly worse compared to that of patients with low CD47 expression [40]. In their study, Li et al. [41] found that CD47 was overexpressed in patients with high-grade serous ovarian carcinoma, and this high CD47 expression was correlated with a poor prognosis. Wang et al. [42] found in their study that CD47 expression was significantly high in ovarian clear cell carcinoma, and this strong expression was correlated with resistance to advanced-stage chemotherapeutics and a poor prognosis.

In our study, it was found that 6 out of 24 chromophobe RCC cases with CD47 staining, 5 out of 7 papillary type 2 RCC cases, all 3 clear cell RCC cases, and 14 out of 18 unclassified RCC cases died within a follow-up period of less than 5 years. A statistically significant relationship was found between the expression rate of CD47 and the survival time in the unclassified RCC cases (*p* = 0.001).

CD47 expression is also associated with tumor metastasis. The activation of osteoclasts can be considered a factor that initiates the metastasis of tumor cells to bones [42,43,44,45]. Recent studies have shown that the SIRP-1/CD47 interaction is associated with macrophage fusion, which plays an important role in the process of osteoclast formation [46,47]. Furthermore, the formation of osteoclasts is influenced by nitric oxide (NO) concentration. Low levels of NO stimulate osteoclast formation, while high levels of NO inhibit it. One study has shown that CD47 can regulate osteoclasts by modulating NO signaling [48].

In their study on breast carcinoma, Bacceli et al. [49] found that CD47 is rarely expressed in non-metastatic tumors, while it is highly expressed in all metastatic cases.

In our study, metastasis was detected in 6 out of 24 chromophobe RCC patients with CD47 staining, in 1 out of 7 papillary type 2 RCC patients, in 2 out of 3 clear cell RCC patients, and in 16 out of 18 unclassified RCC cases. A significant relationship was found between CD47 expression and metastasis in the unclassified RCC cases (*p* = 0.001). In the other groups, no statistically significant difference was observed between CD47 expression and metastasis.

Many studies discussed before showed that immunohistochemical CD47 expression is increased in relation to pathological tumor stage, tumor grade, recurrence, and presence of distant metastases. In our study, increased CD47 expression was determined in unclassified RCCs, which have the worse prognosis compared to the other subtypes, and showed a significant relationship with capsule invasion, metastasis, and patient survival.

CD47 represents a potentially effective and widely applicable target for immune checkpoint-based and cancer immunotherapy. Therefore, a series of inhibitors specifically developed to inhibit the CD47-SIRPα cancer signaling pathway have been created. The use of these agents that inhibit the CD47-SIRPα cancer signaling pathway has been shown to lead to the phagocytosis and elimination of the tumor cells. In this regard, CD47 has been considered a target protein for cancer therapies [23]. In many malignancies, such as acute lymphoblastic leukemia (ALL), acute myeloid leukemia (AML), non-Hodgkin lymphoma (NHL), myeloma, bladder cancer, stomach cancer, glioblastoma, hepatocellular carcinoma, ovarian cancer, breast cancer, colon cancer, head and neck squamous cell carcinoma, and pancreatic neuroendocrine neoplasm (pNEN), a positive response has been observed to treatment regimens based on CD47 blockade [23,25,26,27,28,39,50,51,52,53]. Therefore, the expression of CD47 needs to be researched in various malignant tumors in relation to treatment.

While the most common sites of metastasis for RCC are the liver and the lungs, this tumor can spread to all systems. Metastatic RCC (mRCC) is resistant to chemotherapy, and the average five-year survival rate of patients with mRCC is 71%. However, there is significant variability in patient prognosis [54]. In the past decade, there have been major advances in the treatment of mRCC. Immune checkpoint inhibitors (ICI)-based combination therapies (ICI-ICI or ICI–targeted therapy) are now the main first-line treatments for mRCC, and new combinations are emerging [55]. Since 2005, the United States Food and Drug Administration (FDA) and the European Medicines Agency have approved the anti- VEGF antibody bevacizumab (in combination with interferon), the mTOR inhibitors everolimus and temsirolimus, and the tyrosine kinase inhibitors (TKIs) sorafenib, sunitinib, pazopanib, axitinib, cabozantinib, and lenvatinib to treat mRCC. The treatment for mRCC has entered the immuno-oncology (IO) era with the FDA approval in November 2015 of nivolumab (anti-PD1) monotherapy as a second-line treatment based on the CheckMate 025 study [56]. The presence of a significant relationship between CD47 expression and metastasis in unclassified RCC cases may be promising for the use of anti-CD47 antibodies for this tumor, which has a poor prognosis.

Pembrolizumab, an anti-programmed death1 (PD-1) antibody, was approved in 2021 as an adjuvant treatment for patients with renal cell carcinoma who were at an intermediate-to-high or high risk for recurrence after nephrectomy, with or without the resection of metastatic lesions [57]. The randomized phase 3 KEYNOTE-564 trial revealed a significant improvement in disease-free survival (DFS) with adjuvant pembrolizumab therapy compared to observation alone among patients who underwent nephrectomy for locally advanced renal cell carcinoma (RCC) with a clear cell component [58]. The study’s results led to the FDA approval of single-agent pembrolizumab for the adjuvant treatment of patients with resected ccRCC, at an intermediate–high or high risk of recurrence.

A study conducted in 2015 revealed that CD47 blockade not only is associated with macrophage-mediated phagocytosis but also facilitates the destruction of tumors through T-cell mediated immunogenic pathways [59]. Therefore, the expression status of CD47 needs to be researched in various malignant tumors in relation to treatment. Targeting the CD47-SIRPα signaling system in anticancer therapy is a promising strategy for cancer treatment because this pathway regulates both the innate and the adaptive immune systems [60]. Additionally, experimental studies have shown that in CD47+ tumors treated with anti-CD47 antibodies, the effectiveness of treatments such as chemotherapy and radiotherapy is further enhanced [61]. In light of this information, it is considered that anti-CD47 antibodies could be a treatment option for RCC cases, and more studies are needed in this area.

## 5. Conclusions

Our study is the first to research CD47 expression in benign and malignant renal cell tumors. The identification of a relationship between CD47 expression and distant organ metastasis, prognosis, and 5-year survival suggests that anti-CD47 antibodies could be a treatment modality for RCC patients. There is a need for larger-scale studies in this area to uncover the expression profile of CD47 in renal cell tumors.

## Figures and Tables

**Table 1:** Distribution of patient groups according to RCC subtypes.

Patient Group						
Variable	Clear Cell RCC	Chromophobe RCC	Papillary Type 1 RCC	Papillary Type 2 RCC	Oncocytoma	Unclassified RCC
Age average						
Female	60.1	53.2	75	59.5	62.4	46.6
Male	53.1	58.2	60.7	60.2	59.1	61.8
Gender						
Female	14 (38%)	13	1	4	9	14
		−43%	−3%		−35%	−41%
				−14%		
Male	23 (62%)	17	29	25	17	20
		−57%	−97%		−65%	−59%
				−86%		
T Phase						
T1	12	11	20	13	24	11
T2	13	16	7	13	2	15
T3	11	3	2	1	0	5
T4	1	0	1	2	0	3
Capsule invasion						
Present	8	6	10	8	1	10
Absent	29	24	20	21	25	24
Lymphovascular invasion						
Present	4	3	0	4	0	0
Absent	33	27	30	25	26	34
Distant organ metastasis						
Present	12	6	0	3	0	16
Absent	25	24	30	26	26	18
5-year survey						
Ex	19	6	0	12	0	17
Alive	18	24	30	17	26	17

**Table 2:** Distribution of cases according to staining rates in RCC subtypes.

Patient Group							
CD47 Expression	Clear Cell RCC	Chromophobe RCC	Papillary Type 1 RCC	Papillary Type 2 RCC	Oncocytoma	Unclassified RCC	*p* Value
Negative	34 (92%)	6 (20%)	30 (100%)	22 (76%)	21 (81%)	16 (47%)	
Weak	3 (8%)	23 (77%)	0	6 (21%)	5 (19%)	17 (50%)	0.001
Strong	0	1 (3%)	0	1 (3%)	0	1 (3%)	

**Table 3:** Distribution of cases according to staining prevalence and severity.

	Staining Severity				Staining Prevalence					Number of Positivity
	Negative (0)	Low (1+)	Moderate (2+)	Severe (3+)	0	1+ (<10%)	2+ (10–25%)	3+ (26–50%)	4+ (>50%)	
Clear Cell	34	3	0	0	34	3	0	0	0	3
Chromophobe	6	23	1	0	6	4	5	10	5	24
Papillary Type 1	30	0	0	0	30	0	0	0	0	0
Papillary Type 2	22	6	1	0	22	1	3	3	0	7
Oncocytoma	21	5	0	0	21	1	2	1	1	5
Unclassified	16	17	1	0	16	3	6	4	5	18

## Data Availability

The original contributions presented in the study are included in the article, further inquiries can be directed to the corresponding author.

## References

[1] Hsieh J.J., Purdue M.P., Signoretti S., Swanton C., Albiges L., Schmidinger M., Heng D.Y., Larkin J., Ficarra V. (2017). Renal cell carcinoma. Nat. Rev. Dis. Primers.

[2] Cohen H.T., McGovern F.J. (2005). Renal-Cell Carcinoma. N. Engl. J. Med..

[3] Jemal A., Siegel R., Ward E., Murray T., Xu J., Thun M.J. (2007). Cancer Statistics, 2007. CA Cancer J. Clin..

[4] (1987). Overall Evaluations of Carcinogenicity: An Updating of IARC Monographs Volumes 1 to 42. IARC Monographs on the Evaluation of Carcinogenic Risks to Humans.

[5] Blettner M. (1999). Hormonal Contraception and Post-Menopausal Hormonal Therapy. IARC Monographs on the Evaluation of Carcinogenic Risks to Humans.

[6] Chow W.-H., Gridley G., Fraumeni J.F., Järvholm B. (2000). Obesity, Hypertension, and the Risk of Kidney Cancer in Men. N. Engl. J. Med..

[7] Griffiths D.F.R., Verghese A., Golash A., Kynaston H.G., Matthews P.N., Hart A.J.L., Court J.B. (2002). Contribution of Grade, Vascular Invasion and Age to Outcome in Clinically Localized Renal Cell Carcinoma. BJU Int..

[8] Janzen N.K., Kim H.L., Figlin R.A., Belldegrun A.S. (2003). Surveillance After Radical or Partial Nephrectomy for Localized Renal Cell Carcinoma and Management of Recurrent Disease. Urol. Clin. N. Am..

[9] Lam J.S., Leppert J.T., Figlin R.A., Belldegrun A.S. (2005). Surveillance Following Radical or Partial Nephrectomy for Renal Cell Carcinoma. Curr. Urol. Rep..

[10] Lane B.R., Kattan M.W. (2005). Predicting Outcomes in Renal Cell Carcinoma. Curr. Opin. Urol..

[11] Leach R.E., Miller J.K. (1987). Lateral and Medial Epicondylitis of the Elbow. Clin. Sports Med..

[12] Isenberg J.S., Frazier W.A., Roberts D.D. (2008). Thrombospondins: From Structure to Therapeutics. Cell. Mol. Life Sci..

[13] Gao A.-G., Lindberg F.P., Finn M.B., Blystone S.D., Brown E.J., Frazier W.A. (1996). Integrin-Associated Protein Is a Receptor for the C-Terminal Domain of Thrombospondin. J. Biol. Chem..

[14] Lindberg F.P., Bullard D.C., Caver T.E., Gresham H.D., Beaudet A.L., Brown E.J. (1996). Decreased Resistance to Bacterial Infection and Granulocyte Defects in IAP-Deficient Mice. Science.

[15] Brown E. (2001). Integrin-Associated Protein (CD47) and Its Ligands. Trends Cell Biol..

[16] Liu Y., Merlin D., Burst S.L., Pochet M., Madara J.L., Parkos C.A. (2001). The Role of CD47 in Neutrophil Transmigration. J. Biol. Chem..

[17] Miyashita M., Ohnishi H., Okazawa H., Tomonaga H., Hayashi A., Fujimoto T.-T., Furuya N., Matozaki T. (2004). Promotion of Neurite and Filopodium Formation by CD47: Roles of Integrins, Rac, and Cdc42. Mol. Biol. Cell.

[18] Oldenborg P.-A., Zheleznyak A., Fang Y.-F., Lagenaur C.F., Gresham H.D., Lindberg F.P. (2000). Role of CD47 as a Marker of Self on Red Blood Cells. Science.

[19] Blazar B.R., Lindberg F.P., Ingulli E., Panoskaltsis-Mortari A., Oldenborg P.-A., Iizuka K., Yokoyama W.M., Taylor P.A. (2001). Cd47 (Integrin-Associated Protein) Engagement of Dendritic Cell and Macrophage Counterreceptors Is Required to Prevent the Clearance of Donor Lymphohematopoietic Cells. J. Exp. Med..

[20] Gardai S.J., McPhillips K.A., Frasch S.C., Janssen W.J., Starefeldt A., Murphy-Ullrich J.E., Bratton D.L., Oldenborg P.-A., Michalak M., Henson P.M. (2005). Cell-Surface Calreticulin Initiates Clearance of Viable or Apoptotic Cells through Trans-Activation of LRP on the Phagocyte. Cell.

[21] Chao M.P., Weissman I.L., Majeti R. (2012). The CD47–SIRPα Pathway in Cancer Immune Evasion and Potential Therapeutic Implications. Curr. Opin. Immunol..

[22] Edris B., Weiskopf K., Volkmer A.K., Volkmer J.-P., Willingham S.B., Contreras-Trujillo H., Liu J., Majeti R., West R.B., Fletcher J.A. (2012). Antibody Therapy Targeting the CD47 Protein Is Effective in a Model of Aggressive Metastatic Leiomyosarcoma. Proc. Natl. Acad. Sci. USA.

[23] Willingham S.B., Volkmer J.-P., Gentles A.J., Sahoo D., Dalerba P., Mitra S.S., Wang J., Contreras-Trujillo H., Martin R., Cohen J.D. (2012). The CD47-Signal Regulatory Protein Alpha (SIRPa) Interaction Is a Therapeutic Target for Human Solid Tumors. Proc. Natl. Acad. Sci. USA.

[24] Barclay A.N., van den Berg T.K. (2014). The Interaction Between Signal Regulatory Protein Alpha (SIRPα) and CD47: Structure, Function, and Therapeutic Target. Annu. Rev. Immunol..

[25] Rendtlew Danielsen J.M., Knudsen L.M., Dahl I.M., Lodahl M., Rasmussen T. (2007). Dysregulation of CD47 and the Ligands Thrombospondin 1 and 2 in Multiple Myeloma. Br. J. Haematol..

[26] Jaiswal S., Jamieson C.H.M., Pang W.W., Park C.Y., Chao M.P., Majeti R., Traver D., van Rooijen N., Weissman I.L. (2009). CD47 Is Upregulated on Circulating Hematopoietic Stem Cells and Leukemia Cells to Avoid Phagocytosis. Cell.

[27] Majeti R., Chao M.P., Alizadeh A.A., Pang W.W., Jaiswal S., Gibbs K.D., van Rooijen N., Weissman I.L. (2009). CD47 Is an Adverse Prognostic Factor and Therapeutic Antibody Target on Human Acute Myeloid Leukemia Stem Cells. Cell.

[28] Chao M.P., Alizadeh A.A., Tang C., Myklebust J.H., Varghese B., Gill S., Jan M., Cha A.C., Chan C.K., Tan B.T. (2010). Anti-CD47 Antibody Synergizes with Rituximab to Promote Phagocytosis and Eradicate Non-Hodgkin Lymphoma. Cell.

[29] Ye X., Wang X., Lu R., Zhang J., Chen X., Zhou G. (2018). CD47 as a Potential Prognostic Marker for Oral Leukoplakia and Oral Squamous Cell Carcinoma. Oncol. Lett..

[30] Akel R., Kurban M., Abbas O. (2016). CD47 Expression for in Situ and Invasive Cutaneous Epithelial Lesions. J. Am. Acad. Dermatol..

[31] Park H., Jee S., Bang S., Son H., Cha H., Myung J., Sim J., Kim Y., Paik S., Kim H. (2022). CD47 Expression Predicts Unfavorable Prognosis in Clear Cell Renal Cell Carcinoma After Curative Resection. Diagnostics.

[32] Gudbjartsson T., Hardarson S., Petursdottir V., Thoroddsen A., Magnusson J., Einarsson G.V. (2005). Renal Oncocytoma: A Clinicopathological Analysis of 45 Consecutive Cases. BJU Int..

[33] Delahunt B., Cheville J.C., Martignoni G., Humphrey P.A., Magi-Galluzzi C., McKenney J., Egevad L., Algaba F., Moch H., Grignon D.J. (2013). The International Society of Urological Pathology (ISUP) Grading System for Renal Cell Carcinoma and Other Prognostic Parameters. Am. J. Surg. Pathol..

[34] Amin M.B., Paner G.P., Alvarado-Cabrero I., Young A.N., Stricker H.J., Lyles R.H., Moch H. (2008). Chromophobe Renal Cell Carcinoma: Histomorphologic Characteristics and Evaluation of Conventional Pathologic Prognostic Parameters in 145 Cases. Am. J. Surg. Pathol..

[35] Choudhary S., Rajesh A., Mayer N.J., Mulcahy K.A., Haroon A. (2009). Renal Oncocytoma: CT Features Cannot Reliably Distinguish Oncocytoma from Other Renal Neoplasms. Clin. Radiol..

[36] Fu W., Li J., Zhang W., Li P. (2017). High Expression of CD47 Predicts Adverse Prognosis in Chinese Patients and Suppresses Immune Response in Melanoma. Biomed. Pharmacother..

[37] Zhao H., Wang J., Kong X., Li E., Liu Y., Du X., Kang Z., Tang Y., Kuang Y., Yang Z. (2016). CD47 Promotes Tumor Invasion and Metastasis in Non-Small Cell Lung Cancer. Sci. Rep..

[38] Sudo T., Takahashi Y., Sawada G., Uchi R., Mimori K., Akagi Y. (2017). Significance of CD47 Expression in Gastric Cancer. Oncol. Lett..

[39] Yoshida K., Tsujimoto H., Matsumura K., Kinoshita M., Takahata R., Matsumoto Y., Hiraki S., Ono S., Seki S., Yamamoto J. (2015). CD47 is an Adverse Prognostic Factor and a Therapeutic Target in Gastric Cancer. Cancer Med..

[40] Nagahara M., Mimori K., Kataoka A., Ishii H., Tanaka F., Nakagawa T., Sato T., Ono S., Sugihara K., Mori M. (2010). Correlated Expression of CD47 and SIRPA in Bone Marrow and in Peripheral Blood Predicts Recurrence in Breast Cancer Patients. Clin. Cancer Res..

[41] Li Y., Lu S., Xu Y., Qiu C., Jin C., Wang Y., Liu Z., Kong B. (2017). Overexpression of CD47 Predicts Poor Prognosis and Promotes Cancer Cell Invasion in High-Grade Serous Ovarian Carcinoma. Am. J. Transl. Res..

[42] Kingsley L.A., Fournier P.G.J., Chirgwin J.M., Guise T.A. (2007). Molecular Biology of Bone Metastasis. Mol. Cancer Ther..

[43] Mundy G.R. (2002). Metastasis to Bone: Causes, Consequences and Therapeutic Opportunities. Nat. Rev. Cancer.

[44] Kozlow W., Guise T.A. (2005). Breast Cancer Metastasis to Bone: Mechanisms of Osteolysis and Implications for Therapy. J. Mammary Gland. Biol. Neoplasia.

[45] Roodman G.D. (2004). Mechanisms of Bone Metastasis. N. Engl. J. Med..

[46] Vignery A. (2005). Macrophage Fusion. J. Exp. Med..

[47] Lundberg P., Koskinen C., Baldock P.A., Löthgren H., Stenberg Å., Lerner U.H., Oldenborg P.-A. (2007). Osteoclast Formation Is Strongly Reduced Both In Vivo and In Vitro in the Absence of CD47/SIRPα-Interaction. Biochem. Biophys. Res. Commun..

[48] Uluçkan O., Becker S.N., Deng H., Zou W., Prior J.L., Piwnica-Worms D., Frazier W.A., Weilbaecher K.N. (2009). CD47 Regulates Bone Mass and Tumor Metastasis to Bone. Cancer Res..

[49] Baccelli I., Stenzinger A., Vogel V., Pfitzner B.M., Klein C., Wallwiener M., Scharpff M., Saini M., Holland-Letz T., Sinn H.-P. (2014). Co-Expression of MET and CD47 Is a Novel Prognosticator for Survival of Luminal-Type Breast Cancer Patients. Oncotarget.

[50] Chao M.P., Alizadeh A.A., Tang C., Jan M., Weissman-Tsukamoto R., Zhao F., Park C.Y., Weissman I.L., Majeti R. (2011). Therapeutic Antibody Targeting of CD47 Eliminates Human Acute Lymphoblastic Leukemia. Cancer Res..

[51] Kim D., Wang J., Willingham S.B., Martin R., Wernig G., Weissman I.L. (2012). Anti-CD47 Antibodies Promote Phagocytosis and Inhibit the Growth of Human Myeloma Cells. Leukemia.

[52] Chan K.S., Espinosa I., Chao M., Wong D., Ailles L., Diehn M., Gill H., Presti J., Chang H.Y., van de Rijn M. (2009). Identification, Molecular Characterization, Clinical Prognosis, and Therapeutic Targeting of Human Bladder Tumor-Initiating Cells. Proc. Natl. Acad. Sci. USA.

[53] Zhang M., Hutter G., Kahn S.A., Azad T.D., Gholamin S., Xu C.Y., Liu J., Achrol A.S., Richard C., Sommerkamp P. (2016). Anti-CD47 Treatment Stimulates Phagocytosis of Glioblastoma by M1 and M2 Polarized Macrophages and Promotes M1 Polarized Macrophages In Vivo. PLoS ONE.

[54] Matsuda T., Hori M. (2015). Five-year relative survival rate of kidney and renal pelvis cancer in the USA, Europe and Japan. Jpn. J. Clin. Oncol..

[55] Motzer R.J., Powles T., Burotto M., Escudier B., Bourlon M.T., Shah A.Y., Suárez C., Hamzaj A., Porta C., Hocking C.M. (2022). Nivolumab plus cabozantinib versus sunitinib in first-line treatment for advanced renal cell carcinoma (CheckMate 9ER): Long-term follow-up results from an open-label, randomised, phase 3 trial. Lancet Oncol..

[56] Tucci M., Mandarà M., Giuliani J., Durante E., Buttigliero C., Turco F., Palesandro E., Campisi I., Singh N., Muraro M. (2024). Treatment options in first-line metastatic renal carcinoma: A meta-analysis of 2556 patients treated with immune checkpoint inhibitors-based combinations in randomised controlled trials. Cancer Treat. Rev..

[57] (2024). Highlights of Prescribing Information:Keytruda (Pembrolizumab) Injection, for Intravenous Use.

[58] Choueiri T.K., Tomczak P., Park S.H., Venugopal B., Ferguson T., Symeonides S.N., Hajek J., Chang Y.-H., Lee J.-L., Sarwar N. (2024). Overall Survival with Adjuvant Pembrolizumab in Renal-Cell Carcinoma. N. Engl. J. Med..

[59] Liu X., Pu Y., Cron K., Deng L., Kline J., Frazier W.A., Xu H., Peng H., Fu Y.-X., Xu M.M. (2015). CD47 Blockade Triggers T Cell–Mediated Destruction of Immunogenic Tumors. Nat. Med..

[60] Murata Y., Saito Y., Kotani T., Matozaki T. (2018). CD47-signal Regulatory Protein α Signaling System and Its Application to Cancer Immunotherapy. Cancer Sci..

[61] Dotsikas G., Konowalchuk T., Major P., Kovac P., Ward G., Stewart S., Price G., Elhilali M., Mackillop W. (1987). Cellular Heterogeneity in Normal and Neoplastic Human Urothelium: A Study Using Murine Monoclonal Antibodies. Br. J. Cancer.

